# Healthy Ageing Is Associated with Preserved or Enhanced Nutrient and Mineral Apparent Digestibility in Dogs and Cats Fed Commercially Relevant Extruded Diets

**DOI:** 10.3390/ani11072127

**Published:** 2021-07-17

**Authors:** Sofia Schauf, Jonathan Stockman, Richard Haydock, Ryan Eyre, Lisa Fortener, Jean Soon Park, Anne Marie Bakke, Phillip Watson

**Affiliations:** 1 WALTHAM Petcare Science Institute, Waltham on the Wolds, Leicestershire LE14 4RT, UK; Jonathan.Stockman@liu.edu (J.S.); Richard.Haydock@effem.com (R.H.); Anne.Marie.Bakke@effem.com (A.M.B.); Phillip.Watson@effem.com (P.W.); 2 Royal Canin Pet Health Nutrition Center, Lewisburg, OH 45338, USA; ryan.eyre@royalcanin.com (R.E.); Lisa.Fortener@royalcanin.com (L.F.); jean.soon.park@royalcanin.com (J.S.P.)

**Keywords:** ageing, dog, cat, total dietary fibre, fat digestibility, protein digestibility

## Abstract

**Simple Summary:**

Preventative healthcare and provision of optimal nutrition from early ages is increasing the life expectancy of companion animals today. However, as part of the normal ageing process, changes in the capacity to digest and use dietary nutrients may occur, which could contribute to deficiency in energy and/or essential nutrients. Based on previous studies, an effect of ageing on nutrient digestibility has primarily been observed when feeding low-fat or high-fibre diets. In this study, we evaluated the effect of age on nutrient digestibility using healthy dogs and cats up to 14 years old by feeding diets differing in fat and fibre contents. Older dogs and cats had a preserved nutrient digestibility, in some cases showed an enhanced digestibility of fibre and calcium. Interestingly, older cats had a lower nutrient digestibility with one of the diets, which was unrelated to fat and fibre levels, but was possibly associated with other ingredients in the formulation. Our findings support the use of a wide range of fat and fibre levels in healthy older dogs and cats within the studied ages. However, ingredient sources should be considered in the formulation of senior diets.

**Abstract:**

Age-related changes in gastrointestinal function have been reported in companion animals, but the impact on digestive efficiency remains uncertain. Healthy dogs (*n* = 37; 2.6–14.2 years) received four diets varying in total dietary fibre (TDF; 6–29%, as fed). Healthy cats (*n* = 28; 1–13 years) received four diets with two fat (10–12%; 17–18%) and TDF (9 and 12%) levels. In a crossover design, diets were provided over four consecutive 10-day cycles, including a 4-day faecal collection. Apparent crude protein (CP), ether extract (EE), TDF, calcium (Ca), and phosphorus (P) digestibilities were determined. The effect of age was analysed as a continuous variable in dogs and as differences between adult (1–5 years) and senior (7–13 years) cats. In dogs, EE digestibility was unaffected by age (*p* > 0.10). Dogs of 6–12 years had higher digestibility of CP (*p* = 0.032), TDF (*p* = 0.019), Ca (*p* = 0.019), and P (*p* = 0.024) when fed the 6% TDF diet. Senior cats had greater digestibility of TDF (*p* < 0.01) and Ca (*p* = 0.024) but had lower EE and CP digestibility with one diet (17% fat; 9%TDF) (age, *p* > 0.10; diet × age, *p* < 0.001). Healthy ageing was associated with preserved nutrient digestibility in dogs and cats within the age ranges studied. The effect of ingredient sources in senior cats warrants further investigation.

## 1. Introduction

The life expectancy of dogs and cats continues to increase as a result of improved veterinary care, living conditions, and an increasing number of pet owners providing high-quality nutrition across life stages [1]. Although the concept of life stages is well recognised by pet owners and veterinarians, the age at which pets transition from adulthood to senior is one of debate, partly due to the variability in factors such as breed, body size, and lifestyle. As a result, over the last decade different life stage systems have been proposed to represent physiological and metabolic changes occurring with age. Based on physiological and behavioural changes, the American Animal Hospital Association defines cats as senior when above 10 years [2]. However, earlier changes in body weight (BW), characterised by an increased tendency for cats to become obese when above 7 years and to become underweight when above 12 years, has been demonstrated by Perez-Camargo [3]. A similar decline in the maintenance energy requirements of dogs above 7 years has been reported [4], suggesting an impact of age on energy homeostasis. It is acknowledged that as part of the normal ageing process, the gastrointestinal tract undergoes changes in gut morphology and functionality, which may lead towards a decline in digestive function [5,6]. Gastrointestinal alterations reported with ageing include slower transit time, altered enzymatic activity, impaired circulation, and reduced hydrochloric acid and bile secretion [6,7]. A reduction in duodenal villus surface area and jejunal villus height associated with a greater colonic crypt depth has been reported in dogs above 10 years old [8]. However, there is conflicting evidence regarding the impact of such changes on actual digestive function.

According to a study carried out in adult (<6 years old) and senior (>8 years old) dogs, no significant changes in the apparent digestibility of protein and fat were found, although a trend towards decreased (≤2%) digestibilities for these macronutrients was observed in the older group [4]. However, the diets used in this study were highly digestible (≥84% and ≥94% for protein and fat, respectively) for both age groups, which could have prevented detection of a potential impact of age on digestibility. Using a dog population with a wider age-range (2–3, 8–10, and 16–17 years), Buffington et al. 1989 [9] found no significant differences in protein and fat digestibility associated with age, although dogs above 16 years had a higher variability in nutrient digestibility. The lower protein digestibility of the diets used in this study (<80%) could explain the more variable response found in the older dogs. On the other hand, another study comparing senior (10–12 years) with young adult dogs (1 year) reported a greater apparent digestibility of protein and fat in senior dogs [10].

Several feline studies have demonstrated a decline in fat digestibility in cats above 8 years [4,11,12]. However, protein digestibility in senior cats varied among studies, with reports of no differences [11] or a trend towards decreased apparent digestibility [4] compared to younger adults. The apparent discrepancy between those studies could be partly related to differences in the age range of cat groups being compared, as well as in the nutrient composition of the diets tested. A quadratic relationship between age and the apparent digestibility of fat, protein, and starch was found by Teshima, Brunetto [12]; the digestibility of these nutrients reached a maximum in mature cats (mean of 6 years) decreasing significantly (3–5%) in older cats (mean of 13 years). In addition, the effect of age was primarily observed when cats were fed a low energy diet containing 12% fat and 4% crude fibre (CF; % DM).

The current study aims to evaluate age-related changes in macronutrient and mineral apparent digestibilities in dogs and cats, and to determine whether the observed responses are influenced by variations in dietary fibre and fat content, the main contributors to the digestible energy (DE) content of the diet. We hypothesised that age-related changes in gastrointestinal function of dogs and cats would result in an impaired digestive efficiency, particularly when exposed to diets with a low DE content.

## 2. Materials and Methods

### 2.1. Animals

The study was approved by the WALTHAM Animal Welfare and Ethical Review Body and conducted under the authority of the Animals (Scientific Procedures) Act 1986.

**Figure 1:**
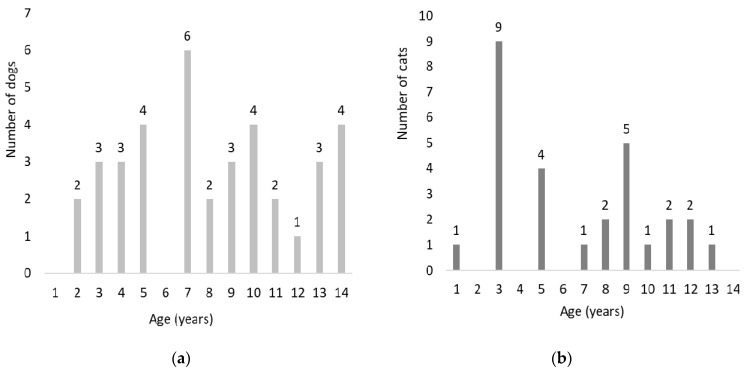
(**a**) Age distribution in the dog population; (**b**) age distribution in the cat population.

The dog population comprised 32 Beagles (25 spayed females, 7 neutered males) with a median age of 7.6 (range 2.6–14.2) years and 5 Brittany dogs (3 neutered males, 2 spayed females) with a median age of 10.4 (range 7.4–10.4) years. For the cat study, twenty-eight domestic short-haired cats (14 neutered male and 14 spayed female cats) with a median age of 6.3 (range 1.2–13.1) years were recruited. Age distribution in dogs and cats is shown in Figure 1. At study entry, median body condition score (BCS) of dogs was 4.0 (range 3.0–6.0). Two Brittany dogs of 7 and 9 years had a BCS of 3, while two 5-year-old Beagles had a BCS of 6. The median BCS of cats was 5.0 (4.0–8.0), with eight between 5 and 12 years scoring from 6 to 8.

Dogs and cats were housed at the Pet Health and Nutrition Centre in Lewisburg (OH, USA). Throughout the study, dogs were pair housed allowing 2 m^2^ per dog, whereas cats were group housed (14 cats per group), except when single-housed during faecal collection in queening cages (2.5 m^2^ floor area and 1.2 m height), allowing them to socialize and exercise for 1 h per day. Dogs were fed twice a day and cats once a day and allowed 30 min per meal. The amount of diet provided for each dog and cat was estimated individually based on previous records of daily energy intakes sufficient to maintain starting BW. Reverse-osmosis water was provided ad libitum throughout the trial. Prior to the study, all included animals were confirmed to be healthy and without a clinical history of dietary sensitivity.

### 2.2. Diets and Apparent Digestibility Protocol

The nutrient and ingredient composition of all diets are provided on as fed basis in Table 1 and Table 2. For the dogs, single batches of four complete and balanced experimental dry extruded diets (Table 1) were formulated at Royal Canin (Aimargues, France) using the same ingredients but providing varying levels of CF and total dietary fibre (TDF): Low-Fibre (1.9% CF, 6.3% TDF), Medium-Fibre (6.3% CF, 13% TDF), High-Fibre (9.2% CF, 16% TDF), and Very-High-Fibre (19% CF and 29% TDF). Crude fibre and TDF levels in the Very-High-Fibre diet approximated the levels found in low energy diets designed for weight loss in dogs (11–23% CF, 18–45% TDF) [13]. Diets differed mainly in the amount of cellulose, which ranged from 0% to 7.2%, 14.5%, and 22%, respectively, replacing corn meal like for like. Additional fibre sources (beet pulp, oligosaccharides, and distiller’s dry grain solubles), fat, and protein levels were kept constant across diets on an as fed basis.

For the cats, single batches of four commercially available dry extruded diets (Premium Original, Special Kitty^®^; Indoor Cat Chicken Flavor, Whiskas^®^; Veterinary Diet Gastrointestinal Fiber Response, Royal Canin^®^; and Grain-Free Adult Salmon & Potato, Nutro^®^) were provided (Table 2). Two diets were low in fat and contained low to moderate levels of CF and TDF: Low-Fat–Low-Fibre diet (LF-LFb; 12% fat, 1.6% CF, 9.0% TDF) and Low-Fat–Moderate-Fibre diet (LF-MFb; 10% fat, 2.9% CF, 12.1% TDF). The other two diets had higher fat levels and contained also low to moderate CF and TDF levels: High-Fat–Low-Fibre diet (HF-LFb; 17% fat, 0.9% CF, 9.5% TDF) and High-Fat–Moderate-Fibre diet (HF-MFb; 18% fat, 3.3% CF, 12% TDF). Fish and plant oil sources were only present in the higher fat diets, whereas beet pulp was only present in the moderate-fibre diets (LF-MFb and HF-MFb). Protein levels were kept constant across the four diets and derived from a mixture of animal- and plant-based sources. Levels of fat and CF in the LF-MFb and the HF-MFb diets approximated the levels provided by a low-energy diet (12.3% fat, 3.4% CF) and high-energy diet (22.4% fat, 3.4% CF), resulting in significant differences in the apparent digestibility of fat, protein, and starch in older cats (13 years) [12].

Dogs and cats were randomly distributed into four dietary groups according to age, sex, BW, and breed (in dogs). Each dietary group received all the diets across four study cycles in a specific counterbalanced dietary order following a Latin square design. Digestibility assays were conducted according to standard guidelines (Association of American Feed Control Officials, 2016). Each study cycle included a 6-day adaptation phase, followed by a 4-day total faecal collection phase. During the adaptation phase, dogs and cats received the same background diet (Medium-Fibre and LF-MFb for dogs and cats, respectively) for 3 days and then transitioned into the new diet over the following 3 days. The new diet was fed to 50% on the first day and to 100% on the second and third days. Each of the test diets introduced was supplemented with titanium dioxide (TiO_2_ powder, 5 g/kg diet) to confirm that faeces represented the test diet only. During collection, faecal samples were collected directly from the floor and faecal consistency for each scored on a 1 to 5 scale using a 17-point internal faecal score scale, whereby a grade of 1 denotes dry and crumbly faeces and a grade of 5 denotes watery faeces. In the dog study, 24 h monitoring was carried out to identify the faeces from each dog and to prevent instances of coprophagia. For the determination of nutrient and mineral apparent digestibility, a sub-sample (around 120 g fresh faeces) was taken from the 4-day pool following homogenisation of the full sample and oven-dried at 70 °C for 72 h prior to analysis. Once dried, the faeces were manually homogenized in a mortar and pestle. A 50 g aliquot of dog and cat faecal samples was sent for dry matter (DM), organic matter (OM), ash, crude protein (CP), fat (as ether extract [EE]), CF, TDF, calcium, and phosphorus analysis at Royal Canin Americas Regional Laboratory (Puslinch, Ontario, Canada). Cat faecal samples were also analysed for starch.

Total tract apparent digestibility (TTAD) of each macronutrient and mineral was calculated as:

(1) $$\mathrm{TTAD}\left(\%\right)=\frac{4-\mathrm{day}\;\mathrm{nutrient}\;\mathrm{intake}\left(g\right)\;-\;4-\mathrm{day}\;\mathrm{nutrient}\;\mathrm{faecal}\;\mathrm{excretion}\left(g\right)\;}{4-\mathrm{day}\;\mathrm{nutrient}\;\mathrm{intake}\left(g\right)}\times 100$$

The BW of dogs and cats was recorded before study start and weekly throughout the study. The BCS was assessed at study start and study end according to a 1–9 point scale [14].

### 2.3. Analytical Methods

Food and dried faeces were ground in a cutting mill with a 1-mm sieve and analysed for DM, ash, CP, EE, CF, and TDF according to standard methods (AOAC International, 1995 and 2005) [15,16]. Crude fibre was only determined in food samples. Crude protein was determined according to Dumas method (AOAC no. 968.02). Fat content (as EE) was measured by acid hydrolysis and ether extraction (AOAC no. 954.02). Total dietary fibre content was analysed by an enzymatic method (AOAC 991.43). The starch content was analysed in food and faecal samples of cats by an enzymatic method (AOAC 996.11). Measurement of Ca and P in food and faeces was carried out by atomic absorption spectroscopy preceded by microwave digestion [17]. Gross energy (GE) content was determined using bomb calorimetry [18] in the cat diets and in faecal samples of dogs. The GE content of the dog diets was estimated based on the analysed chemical composition according to NRC (2006) [19].

### 2.4. Statistical Analysis

As the dogs were dispersed across the age ranges, the effect of age was analysed using a generalised additive mixed model, including age as a continuous variable. The model contained a smooth fit describing how the apparent digestibility of each diet changed across the age range, the fixed effect of diet and the random effect of dog. ANOVA was used to test whether the apparent digestibility of nutrients and minerals changed as dogs aged across diets. As cats were equally distributed in distinct age groups (1–5 and 7–13 years) and the majority of younger cats were 3 years, the effect of age was analysed as the differences between age groups using a linear mixed-effects model, including the fixed effects of diet, age, diet by age interaction, and a random effect of cat to account for repeated measures. ANOVA was used to investigate whether the effect of age differed across diets. Statistical analyses were performed using R version 3.3.3 and the *lme4*, *multcomp* and *mgcv* libraries. All contrasts were adjusted using the Benjamini-Hochberg method or Tukey HSD to maintain a false discovery rate of 5%.

## 3. Results

A 13-year-old Beagle was removed from the last two study cycles in which the Very-High and High-Fibre diets were due to be tested, respectively, due to weekly BW loss of 3.9% alongside gastrointestinal symptoms while on the Very-High-Fibre diet. Three cats (a 5-year-old and two cats 9 and 10 years old) displayed poor acceptability of the LF-MFb and were removed from that specific cycle.

### 3.1. Nutrient Composition of Diets on an Energy Basis

In the dog study, there was a 10- and 4.7-fold difference in CF and TDF content between the Low-Fibre and the Very-High-Fibre diets, resulting in a 41% difference in their ME content (Table 1). Due to the decreased ME content with increasing fibre levels, protein and fat contents on an ME basis were higher in the Very-High-Fibre diet (96 g CP/Mcal and 46 g EE/Mcal) compared with the High-Fibre (78 g CP/Mcal and 40 g EE/Mcal), Medium-Fibre (72 g CP/Mcal and 41 g EE/Mcal), and Low-Fibre diet (66 g CP/Mcal and 38 g EE/Mcal).

In the cat diets, differences in fat and fibre levels resulted in an 11% difference in the ME content (Table 3). On an ME basis (per Mcal), protein and starch were higher in the two low fat diets (LF-LFb: 92 g CP and 72 g starch; LF-MFb: 91 g CP and 74 g starch) compared with HF-LFb (80 g CP and 62 g starch) and HF-MFb (87 g CP and 50 g starch).

### 3.2. Diet Intake, Body Weight, and Body Condition Score

The median daily energy intake of dogs was 97.9 (range 61.0–153) kcal/kgBW^0.75^. A significant effect of age on energy intake was not observed (*p* = 0.731). Within the same age range (7–10 years), Brittany dogs had 1.5-fold higher daily energy intake than Beagles (Table 3). Throughout the study, the BW of dogs remained within a 10% variation and most dogs maintained their starting BCS, except for one Beagle (7.4 years) and one Brittany (10.4 years), whose BCS increased from 5 to 6.

Senior cats had a lower (*p* = 0.006) daily energy intake (median 60.1, range 41.1–98.8 kcal/kg^BW0.711^) compared to adults (median 70.3, range 46.2–91.2 kcal/kgBW^0.711^) (Table 4). The BW of cats remained within 10% variation, but three cats (one 3-year-old and two cats of 10 and 13 years) with a starting BCS of 4 were scored as underweight (BCS = 3) at the end of the study.

### 3.3. Nutrient Apparent Digestibility in Dogs

**Figure 2:**
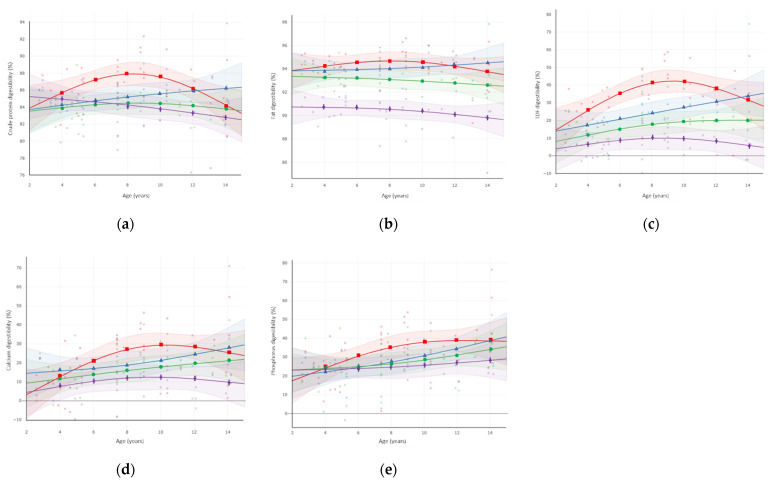
Apparent digestibility (%) of (**a**) crude protein; (**b**) fat; (**c**) total dietary fibre (TDF); (**d**) calcium; (**e**) phosphorus as a function of age in dogs fed the Low-Fibre (red line; square), Medium-Fibre (blue line; triangle), High-Fibre (green line; circle), and Very-High-Fibre (purple line; cross) diets. Shaded areas denote 95% confidence intervals.

Daily DM intake, macronutrient, and mineral apparent digestibilities, faecal DM and consistency score for each diet and across all ages, as well as *p*-values for diet and age effects on these parameters are given in Table 5. The effect of age on nutrient digestibilities for each diet is specifically illustrated in Figure 2a–e.

A significant effect of diet was consistently observed for all parameters assessed (*p* < 0.05). Food adjustments applied to maintain daily energy intake throughout the trial resulted in significant differences in DM intake for the different diets (*p* < 0.001). Dry matter, GE, and TDF apparent digestibilities decreased significantly (*p* < 0.001 for all) with each increase in dietary TDF, and the same trend was observed for all the other nutrients. Feeding the Very-High-Fibre diet resulted in a lower apparent digestibility of fat (−3.9%, *p* < 0.001), CP (−2.3%, *p* = 0.001), TDF (−28.5%, *p* < 0.001), Ca (−12%, *p* = 0.002), and P (−7.5%, *p* = 0.022) compared with the Low-Fibre diet. Mean faecal DM was higher (*p* < 0.01), while faecal consistency score was lower (*p* < 0.001) for the Very-High-Fibre diet in relation to the other three diets.

A significant effect of age on daily DM intake was not observed (*p* > 0.10). Fat apparent digestibility did not significantly differ (*p* > 0.10) at any age for any of the diets (Figure 2b). Any effect of age on nutrient digestibility was limited to the Low-Fibre diet (Table 4), this response affecting similarly both dog breeds (Figure S1a–d) and gender (Figure S2a–d). While on this diet, dogs of 6–10 years had higher (*p* = 0.032) CP digestibility, while dogs of 8–12 years had higher TDF (*p* = 0.019), Ca (*p* = 0.019), and P (*p* = 0.024) digestibilities compared to younger and older dogs (Figure 2a,c–e). An effect of age on faecal DM or consistency scores was not observed with any diet (*p* > 0.10), faecal scores remaining between 2 and 2.5 across diets.

### 3.4. Nutrient Apparent Digestibility in Cats

The effects of diet and age-group on daily DM intake and nutrient apparent digestibilities are shown in Table 6.

A significant effect of diet was observed for all parameters investigated (*p* < 0.05) with the exception of TDF digestibility (*p* > 0.10). Starch digestibility was highest (>99%) for all diets, followed by fat and CP. Calcium digestibility was lowest, with negative numbers often observed. Feeding the HF-LFb diet resulted in a lower faecal DM content and a higher faecal consistency score (*p* < 0.001 for both) compared with the other three diets, faecal scores remaining between 2.0 and 2.9 across diets.

Senior cats displayed a lower DM intake compared to adults (*p* = 0.012), with significant differences observed when fed the two low-fibre (LF-LFb −15%, *p* = 0.023; HF-LFb −39%, *p* < 0.001) diets. A general effect of age on DM, CP, fat, starch and P digestibilities were not observed (*p* > 0.10) and any specific difference between adult and senior cats was only found when fed the LF-MFb and/or HF-LFb diets (age-group × diet interactions, *p* ≤ 0.001) for DM, CP, fat, and starch. Senior cats fed the HF-LFb diet had a lower digestibility of CP (−4.1%, *p* = 0.012), fat (−7.2%, *p* < 0.001), and starch (−0.2%, *p* = 0.001) compared to adults, with mean CP and fat digestibilities dropping to 78–79%. On the other hand, adult cats had lower DM (−5.8%, *p* = 0.002) and CP (−5.1%, *p* = 0.012) digestibilities compared to senior cats when fed the LF-MFb diet. Senior cats consistently displayed higher TDF (+12.7%, *p* = 0.002) and Ca (+9.9%, *p* = 0.024) apparent digestibilities, whereas P digestibility did not significantly differ between age-groups (*p* > 0.10). No significant differences in faecal DM and consistency score were observed between age-groups across diets (*p* > 0.10).

## 4. Discussion

In this study, we assessed the effect of age on nutrient total tract apparent digestibility in dogs and cats in age ranges of 2–14 and 1–13 years, respectively, in response to diets with varying digestible energy content. Across both species, diet composition had more consistent impacts than age, while detected effects of age varied depending on diet. The effect of age on nutrient digestibility in both species and the influence of diet on that response is here discussed.

In dogs, apparent nutrient digestibility was generally well preserved up to an age of 14 years, with middle-aged dogs up to 10 years displaying increases in protein, Ca, P, and TDF digestibilities when fed a low fibre diet with a highly digestible energy content. Fat digestibility was impacted least by age, as observed in previous studies [4,9] reporting data in dogs up to 15 or 17 years. As in the current study, higher protein digestibility was also observed by Sheffy et al. 1985 [10] in 10–12-year-old dogs compared with 1-year-old dogs when feeding diets containing slightly higher crude fibre levels (3.4–5.4% on as fed basis). This could be partly related to an intestinal adaption to ageing, as demonstrated in rodents. In this species, a 3-fold increase in specific and total aminopeptidase activity (per mg protein and per cm intestine length, respectively) was detected in the proximal ileum of older rats [20].

In addition, dogs of 8–12 years in this study coped with high TDF levels at least as well as younger dogs and even showed an enhanced TDF digestibility when fed the Low-Fibre diet, which had a higher TDF digestibility compared to the other three diets. This finding may be indicative of an adaptative process occurring with age resulting in a higher capacity to ferment more fermentable fibres, such as those provided in this diet. The parallel increase in Ca and P apparent digestibilities observed in dogs of 8–12 years while on the Low-Fibre diet could also have been attributable to a higher fermentation of fibre in these dogs. Based on previous observations in rats [21], a decrease in intestinal pH induced by fibre fermentation enhances Ca absorption by increasing the concentration of ionized Ca and its absorption via passive diffusion. However, the apparent digestibility of Ca and P were similar to previous values reported in dogs [22,23]. To the best of our knowledge, there is no direct evidence reported in the literature on the effect of age on fibre fermentation. Although anatomical changes in the hindgut of senior dogs (12 years) compared to young dogs (1.2 years) have been reported [8], accompanying changes in fermentative end-products are not consistent among studies [8,24], likely due to differences in age ranges, diets, and other animal factors. As fermentation end-products were not measured in the current study, age and diet effects on TDF apparent digestibility should be interpreted cautiously.

In cats, consistent effects of age were limited to TDF and Ca digestibilities, which increased in senior cats, as was also observed in older dogs when fed the Low-Fibre diet. No consistent effects of age on DM, fat, protein or starch apparent digestibilities were demonstrated, although the differing responses of adult and senior cats with the LF-MFb and HF-LFb diets suggest an age-related effect on the digestibility of certain types of ingredients delivering protein and fat, which may help explain the conflicting findings among earlier studies [3,4,11,12,25]. Two of these studies showed a decline in fat digestibility occurring with age [4,12], which according to one study [25] was accompanied by subnormal serum cobalamin, and therefore attributed to subclinical chronic gastrointestinal disease. A remarkable decline in the apparent digestibility of protein (<77%) or starch (<96%) has been primarily found in cats at or above 13 years [3,12]. Therefore, it is possible that the lower age and relatively low number of cats above 10 years old in the study reported herein (5/14) may have prevented detection of a more consistent effect of age.

Similar to the outcomes in dogs, higher TDF and Ca apparent digestibilities were observed in senior cats compared to adults, this finding being consistent across diets containing 9–12% TDF and different fibre sources. As in the case of dogs, Ca apparent digestibility remained within values previously reported in cats [26].

In the dog study, increasing the dietary cellulose while keeping inclusion of beet pulp and distillers’ dry grain solubles constant caused an increase in the relative proportions of insoluble to soluble fibre. The higher relative amount of fermentable fibre in the Low-Fibre diet, with no cellulose added, could explain the higher TDF apparent digestibility observed in dogs on this diet. Higher digestibility of beet pulp compared to cellulose has previously been reported in dogs [27]. Both the lack of cellulose and the increased relative amount of soluble fibre in the Low-Fibre diet could explain the comparatively higher apparent digestibility of Ca and P observed in dogs while on this diet. Cellulose addition at levels between 4 and 12% has been shown to decrease Ca apparent digestibility in a dose-dependent manner in pigs [28], while Ca absorption has been correlated with fibre fermentability in rat studies using structural (oat fibre and external pea fibre) and more soluble fibres (beet fibre and internal pea fibre) at 13% or 26% TDF [21]. Fermentable fibre supplementation using oligofructose and inulin has been also associated with increased Ca absorption in dogs [29]. On the other hand, the stable inclusion level of soluble fibre sources in the dog diets containing TDF levels of 6, 13, 16, and 29% could partly explain why minimal changes in mean fat and protein digestibility were observed, with values remaining above 84% and 90% across diets, respectively. It is recognized that dietary fibre may impact nutrient digestibility through an increased passage rate, decreased pH, dilution of enzymes in the chyme, and/or by binding nutrients [30]. However, the impact of fibre on protein digestion in dogs seems to be quite variable [30,31], and any negative impact of fibre on protein and fat digestibility has been mainly ascribed to fermentable fibre [27]. This has been attributed to an enhanced microbial growth and synthesis of microbial protein and lipid, as well as to decreased ammonia absorption in the hindgut, resulting in increased faecal N and fat excretion [32]. The minimal impact of cellulose addition on protein and fat digestibility reported in the current study could have contributed to the lack of age-related changes when testing the three diets with higher TDF levels. In a previous study in dogs [33], a lower protein and fat digestibility was reported in 10-year old dogs compared with 3-year old in response to beet pulp supplementation, although not in response to non-fermentable fibre (sugar cane) supplementation. It is also plausible that the higher protein and fat content on an energy basis of the higher fibre diets could have contributed to an increased apparent digestibility of protein and fat with these diets.

In the cat arm of the study, the differing ingredient sources, the unknown inclusion levels of ingredients in the diets as well as potential dietary differences in kibble shape and processing conditions make a full interpretation of the results obtained difficult. However, macronutrient digestibility was generally highest in both adult and senior cats fed the HF-MFb diet, which had three animal-based ingredients among the first five ingredients listed compared to one or two for the other diets. This suggests that most cats, including senior cats, can use animal-based ingredients to a greater extent than plant-based ingredients. This is also supported by the similar or even higher apparent digestibility of protein observed in senior cats compared to adults when fed the LF-LFb and LF-MFb diets, respectively, both of which contained poultry by-product meal as the first ingredient. On the other hand, the lower protein apparent digestibility of senior cats when fed the HF-LFb may suggest a lower tolerance to specific ingredients used in this diet or to a combination of them. For example, a lower protein digestibility of plant-based protein sources containing antinutritional factors has been shown in older compared to younger rats [34]. This finding was corroborated by the lower fat digestibility (78%) observed in senior cats when fed the HF-LFb diet, which contained a similar total fat level than the HF-MFb (17–18%) but from different ingredient sources. Although fish oil, rich in polyunsaturated fatty acids (PUFA), was the main fat source in the HF-LFb diet, certain PUFA may have a lower hydrolysis rate [35], which is related to the location of the double bond and molecular structure [36].

Some limitations of the study included the short duration of faecal collections, which prevented assessment of any long-term implications of fibre addition on fermentative activity as well as on mineral digestibility. The higher TDF and Ca apparent digestibility reported in our study in older dogs and cats when fed low to moderate TDF levels supports further work to better explore the effect of age on these parameters over the longer term. In both studies, but especially in the cat study, the number of animals above the age of 10 was relatively low, and this could have prevented a major effect of age to be detected. The impact of ingredient sources on nutrient digestibility in the senior population, particularly in cats, warrants further investigation.

## 5. Conclusions

The current findings indicate a preserved digestive capacity in healthy dogs up to 14 years old when fed low energy diets containing up to 29% TDF, provided primarily as cellulose (22%), and in healthy cats up to 13 years when fed low energy diets low in fat (10–12%) and with low to moderate TDF (9.0–12%). In older cats, macronutrient apparent digestibility seemed to be more dependent on dietary ingredient composition than on macronutrient level, with certain ingredient combinations having a negative impact on digestibility. In both species, older animals had an increased TDF apparent digestibility when offered low to moderate TDF, which was associated with an increased apparent digestibility of calcium. Our data support the use of a wide range of fat and fibre levels for the formulation of senior diets. However, the potential impact of ingredient sources on macronutrient digestibility and of fermentable fibre addition on calcium and phosphorus absorption in older dogs and cats warrants further investigation.

## Figures and Tables

**Table 1:** Nutrient composition and energy content on as fed basis of the diets used in dogs.

Diet Composition	Low-Fibre ^1^	Medium-Fibre ^2^	High-Fibre ^3^	Very-High-Fibre ^4^
Chemical analysis (g/100 g)	-	-	-	-
Moisture	9.40	9.50	9.50	9.80
Ash	6.11	6.28	6.46	6.63
Protein	25.9	25.1	26.3	26.1
Fat	14.5	14.4	13.5	13.0
Saturated (% fatty acids)	33.7	33.5	32.9	33.3
Monounsaturated (% fatty acids)	46.3	46.3	45.1	45.2
Polyunsaturated (% fatty acids)	20.0	20.2	22.0	21.5
Crude fibre (CF)	1.90	6.30	9.20	19.5
Total dietary fibre (TDF)	6.30	13.1	16.1	29.3
Calcium	1.17	1.29	1.21	1.20
Phosphorus	0.80	0.84	0.79	0.76
Ca: P ratio	1.46	1.54	1.53	1.58
Starch gelatinization (%)	91.2	97.3	95.8	93.0
Energy content	-	-	-	-
GE ^5^ (Mcal/kg)	4.65	4.62	4.58	4.53
GE ^6^ digestibility (%)	88.2	81.3	79.7	65.7
DE ^7^ (Mcal/kg)	4.11	3.75	3.66	2.99
ME ^8^ (Mcal/kg)	3.83	3.49	3.38	2.71

DM, dry matter; GE, gross energy; ME, metabolizable energy; NFE, nitrogen free extractives. ^1^ Poultry meal (22.9%), corn (18.8%), corn flour (21.8%), cellulose (0%), wheat (6.9%), lard (6.3%), pork meal(4.0%), L-arabinose syrup (3.2%), beet pulp (1.98%), fish oil (1.30%), brewers dried yeast (0.99%), refined soya oil (0.99%), distiller’s dry grain solubles (0.69%), mineral and vitamin premix (0.55%), salt (0.5%), premix oligosaccharides (0.38%), potassium chloride (0.45%), potassium sorbate (0.35%), premix antioxidants (0.30%), choline (0.23%), mannan oligosaccharides (0.20%), vitamin E (0.10%), methionine (0.10%). ^2^ Poultry meal (23.7%), corn (18.9%), corn flour (13.5%), cellulose (7.22%), wheat (6.95%), lard (6.4%), pork meal(4.0%), L-arabinose syrup (3.2%), beet pulp (1.99%), fish oil (1.31%), brewers dried yeast (0.99%), refined soya oil (0.99%), distiller’s dry grain solubles (0.69%), mineral and vitamin premix (0.56%), salt (0.5%), premix oligosaccharides (0.38%), potassium chloride (0.48%), potassium sorbate (0.35%), premix antioxidants (0.30%), choline (0.24%), mannan oligosaccharides (0.20%), vitamin E (0.10%), methionine (0.10%). ^3^ Poultry meal (24.7%), corn (19.0%), corn flour (7.5%), cellulose (14.5%), wheat (7.0%), lard (6.4%), pork meal (4.0%), L-arabinose syrup (3.2%), beet pulp (1.99%), fish oil (1.31%), brewers dried yeast (1.00%), refined soya oil (1.00%), distiller’s dry grain solubles (0.70%), mineral and vitamin premix (0.56%), salt (0.5%), premix oligosaccharides (0.38%), potassium chloride (0.51%), potassium sorbate (0.35%), premix antioxidants (0.30%), choline (0.24%), mannan oligosaccharides (0.20%), vitamin E (0.10%), methionine (0.10%). ^4^ Poultry meal (25.6%), corn (18.5%), corn flour (0.0%), cellulose (22.0%), wheat (7.1%), lard (6.3%), pork meal(4.0%), L-arabinose syrup (3.2%), beet pulp (2.00%), fish oil (1.30%), brewers dried yeast (1.00%), refined soya oil (1.00%), distiller’s dry grain solubles (0.70%), mineral and vitamin premix (0.56%), salt (0.5%), premix oligosaccharides (0.38%), potassium chloride (0.53%), potassium sorbate (0.35%), premix antioxidants (0.30%), choline (0.25%), mannan oligosaccharides (0.20%), vitamin E (0.10%), methionine (0.10%). ^5^ GE = (5.7 × g protein) + (9.4 × g fat) + (4.1 × g [NFE + CF]); NFE = Organic matter-(moisture + ash + protein + fat). ^6^ Calculated using NRC 2006 predictive equation based on CF content: (92 − 1.43 × CF [% DM]) for diets with CF < 8% DM, or based on TDF content: (96.6 − 0.95 × TDF [% DM]) for diets with CF > 8% DM. ^7^ Digestible energy (DE) = GE × GEd/100. ^8^ Calculated by subtracting 1.04 kcal per g crude protein (DM) to the DE content (DE = GE × GEd/100).

**Table 2:** Nutrient composition and energy content on as fed basis of the diets used in cats.

Diet Composition	LF-LFb ^1^	LF-MFb ^2^	HF-LFb ^3^	HF-MFb ^4^
Chemical analysis (g/100 g)	-	-	-	-
Moisture	4.10	5.90	5.40	5.20
Ash	10.4	6.70	7.10	7.90
Protein	34.7	33.6	33.0	35.2
Fat	12.0	10.1	17.0	17.7
Saturated (%fatty acids)	31.7	35.3	31.3	27.9
Monounsaturated (%fatty acids)	46.6	45.2	46.8	44.1
Polyunsaturated (%fatty acids)	21.8	19.5	21.9	27.9
Starch	27.5	27.4	25.5	20.1
Crude fibre (CF)	1.60	2.90	0.90	3.30
Total dietary fibre (TDF)	9.00	12.1	9.50	12.2
Calcium	2.81	1.22	1.02	1.34
Phosphorus	1.67	1.01	0.96	1.10
Ca: P ratio	1.68	1.20	1.06	1.22
Energy content	-	-	-	-
GE ^5^ (Mcal/kg)	4.69	4.68	5.06	5.05
GE ^6^ digestibility (%)	86.4	85.2	87.1	84.8
DE ^7^ (Mcal/kg)	4.05	3.99	4.40	4.28
ME ^8^ (Mcal/kg)	3.79	3.71	4.11	4.03

DM, dry matter; GE, gross energy; LF-LFb, Low Fat-Low Fibre; LF-MFb, Low Fat-Moderate Fibre; HF-LFb, High Fat-Low Fibre; HF-MFb, High Fat-Moderate Fibre. ^1^ Poultry by product meal, ground whole corn, ground whole wheat, meat and bone meal, soybean meal, animal fat, yeast, taurine, vitamin and minerals (calcium provided as calcium pantothenate and calcium iodate; phosphorus provided as phosphorus oxide). ^2^ Poultry by-product meal, ground yellow corn, ground wheat, soybean meal, corn gluten meal, dried plain beet pulp, natural chicken flavour, animal fat, powdered cellulose, taurine, yucca schidigera extract, vitamin and minerals (calcium provided as calcium carbonate and d-calcium pantothenate; phosphorus source not specified). ^3^ Brewers rice, chicken by-product meal, chicken fat, corn, corn gluten meal, wheat gluten, psyllium seed husk, chicory, egg product, fish oil, grain distillers dried yeast, fructooligosaccharides, vegetable oil, hydrolysed yeast, taurine, vitamin and minerals (calcium provided as calcium sulphate, D-calcium pantothenate and calcium iodate; phosphorus provided as potassium phosphate). ^4^ Salmon, Chicken Meal, Pea Protein, Chicken Fat, Dried Potatoes, Potato Starch, Peas, Dehydrated Alfalfa Meal, Potato Protein, Dried Plain Beet Pulp, Soybean Oil, Taurine, vitamins and minerals (calcium provided as calcium pantothenate and phosphorus provided as L-Ascorbyl−2-Polyphosphate). ^5^ Determined by bomb calorimetry. ^6^ Calculated based on NRC 2006 predictive equation: GEd = 87.9 − 0.88 × CF [% DM]). ^7^ Digestible energy (DE) = GE × GEd/100. ^8^ Calculated by subtracting 0.77 kcal per g crude protein (DM) to the DE content (DE = GE × GEd/100).

**Table 3:** Description of the dog population.

Dog Population	Study Start	Study End
	Median (min, max)	Median (min, max)
Body weight, kg	10.4 (7.8, 22.0)	10.2 (7.5, 22.3)
Beagle	10.2 (7.8, 13.8)	10.0 (7.5, 14.0)
Brittany	16.1 (13.0, 22.4)	16.9 (13.0, 22.4)
Female (F)	10.0 (7.8, 18.0)	9.6 (7.5, 18.7)
Male (M)	12.1 (10.2, 22.4)	12.0 (10.2, 22.3)
Body condition score	4.0 (3.0, 6.0)	4.0 (3.0, 6.0)
3 (underweight)	1 Brittany/1 Beagle/2 M	1 Brittany/1 Beagle/2 M
4–5 (ideal)	29 Beagles/4 Brittany/25 F/8 M	27 Beagles/3 Brittany/23 F/7 M
6 (overweight)	2 Beagles/2 F	3 Beagles/1 Brittany/3 F/1 M
Energy intake (kcal/kgBW^0.75^)	-	-
Beagles (2–5 years)	95.1 (65.7, 144)	98.0 (65.7, 131)
Beagles (7–10 years)	91.8 (72,7, 119)	90.8 (74.1, 115)
Brittany (7–10 years)	143 (116, 150)	144 (117, 151)
Beagles (10–14 years)	96.8 (73.9, 151)	94.6 (75.3, 151)
Females (2–5 years)	95.1 (65.7, 120.2)	98.1 (65.7, 130.9)
Females (7–14 years)	93.7 (72.7, 116.0)	93.3 (74.2, 117.3)
Males (7–14 years	111.0 (77.3, 150.2)	110.6 (75.2, 150.7)

**Table 4:** Description of the cat population.

Cat Population	Study Start	Study End
	Median (min, max)	Median (min, max)
Body weight, kg	4.2 (2.6, 5.5)	4.0 (2.5, 5.3)
Cats (1–5 years)	3.9 (3.2, 5.0)	3.8 (3.3, 4.8)
Cats (7–13 years)	4.5 (2.6, 5.5)	4.4 (2.5, 5.3)
Body condition score	5.0 (4.0, 8.0)	4.0 (3.0, 8.0)
3 (underweight)	*n* = 0	*n* = 3
4–5 (ideal)	*n* = 20	*n* = 18
6–7 (overweight)	*n* = 7	*n* = 6
8 (obese)	*n* = 1	*n* = 1
Energy intake (kcal/kgBW^0.711^)	-	-
Cats (1–5 years)	70.5 (46.4, 70.5)	74.3 (61.2, 91.3)
Cats (7–13 years)	58.8 (42.8, 96.9)	61.9 (48.0, 82.9)

**Table 5:** Mean and 95% confidence intervals (CI) for dry matter (DM) intake, apparent digestibility of DM and nutrients, faecal DM and consistency score in dogs. *p*-Values indicate effects of diet within dogs of 2–14 years and of age within each diet.

Performance Parameters	Low-Fibre		Medium-Fibre		High-Fibre		Very-High-Fibre		*p* (*Diet*) ^1^
	Mean (95% CI)	*p* (*Age*)	Mean (95% CI)	*p* (*Age*)	Mean (95% CI)	*p* (*Age*)	Mean (95% CI)	*p* (*Age*)	
DM intake (g/kgBW^0.75^)	26.8 (24.8,28.8)	0.670	28.9 (26.9,30.9)	0.670	30.7 (28.7,32.7)	0.670	33.4 (31.4,35.4)	0.670	<0.001
Digestibility (%)	-	-	-	-	-	-	-	-	-
Dry matter	86.3 (84.6,87.9) ^a^	0.175	79.6 (77.9,81.3) ^b^	0.286	75.3 (73.6,77.0) ^c^	0.750	60.0 (58.3,61.7) ^d^	0.807	<0.001
Protein	86.4 (85.3,87.5) ^a^	0.032	85.1 (84.0,86.3) ^ab^	0.359	84.2 (83.0,85.3) ^b^	0.822	84.1 (83.0,85.2) ^b^	0.807	<0.001
Fat	94.3 (93.7,94.9) ^a^	0.475	94.1 (93.4,94.7) ^a^	0.697	93.0 (92.4,93.7) ^b^	0.750	90.4 (89.8,91.0) ^d^	0.807	<0.001
TDF	36.7 (29.2,40.1) ^a^	0.019	24.3 (18.9,29.7) ^b^	0.082	16.6 (11.3,21.9) ^c^	0.714	8.19 (2.75,13.6) ^d^	0.807	<0.001
Calcium	22.4 (16.8,28.0) ^a^	0.019	20.2 (14.6,25.9) ^a^	0.299	16.0 (10.6,21.4) ^ab^	0.714	10.3 (4.85,15.9) ^b^	0.807	<0001
Phosphorus	32.8 (27.6,38.0) ^a^	0.024	28.5 (23.3,33.7) ^ab^	0.082	27.5 (22.4,32.7) ^ab^	0.714	25.3 (20.1,30.5) ^b^	0.807	0.037
Gross energy	90.3 (89.0,91.6) ^a^	0.224	84.3 (82.9,85.6) ^b^	0.299	80.1 (78.8,81.4) ^c^	0.750	67.6 (66.1,69.1) ^d^	0.807	<0.001
Faecal DM (%)	34.5 (33.6,35.5) ^c^	0.475	37.8 (36.8,38.7) ^b^	0.615	37.2 (36.2,38.2) ^b^	0.750	39.2 (38.2,40.2) ^a^	0.975	<0.001
Faecal score ^2^	2.29 (2.23,2.35) ^a^	0.409	2.18 (2.12,2.24) ^c^	0.878	2.24 (2.18,2.30) ^b^	0.292	2.04 (1.98,2.10) ^d^	0.643	<0.001

TDF, total dietary fibre. ^1^ Different superscript letters (^a,b,c,d^) within each row denote significant (*p* < 0.05) differences between diets. ^2^ Based on a 1–5 scoring system, with increasing values indicating increasing softness in stool quality.

**Table 6:** Mean and 95% confidence intervals (CI) for dry matter (DM) intake, apparent digestibility of DM and nutrients, faecal DM and consistency score in adult (1–5 years) and senior (7–13 years) cats.

Performance Parameters	Age	Diet				*p*-Values		
		LF-LFb	LF-MFb	HF-LFb	HF-MFb	Age	Age × Diet ^1^	Diet
DM intake (g/kgBW^0.711^)	Adult	18.8 (17.3,20.3) ^a^	16.4 (14.9,18.0)	18.6 (17.1,20.1) ^a^	17.6 (16.1,19.2)	0.012	<0.001	0.009
	Senior	16.3 (14.8,17.8) ^b^	16.2 (14.6,17.7)	13.3 (11.8,14.8) ^b^	16.3 (14.8,17.9)			
Digestibility (%)	-	-	-	-	-	-	-	-
Dry matter	Adult	71.4 (69.4,73.5)	70.7 (68.6,72.8) ^b^	75.8 (73.7,77.8)	73.3 (71.3,75.3)	0.201	0.001	0.032
	Senior	72.6 (70.6,74.7)	76.5 (74.3,78.6) ^a^	73.7 (71.6,75.7)	76.1 (74.1,78.1)	-	-	-
Crude protein	Adult	78.4 (76.6,80.1)	75.8 (74.0,77.7) ^b^	82.7 (80.9,84.5) ^a^	84.3 (82.5,86.1)	0.995	<0.001	<0.001
	Senior	79.0 (77.2,80.8)	80.9 (79.0,82.8) ^a^	78.6 (76.8,80.4) ^b^	84.9 (83.1,86.7)	-	-	-
Fat	Adult	85.8 (84.0,87.7)	83.6 (81.6,85.4)	85.1 (83.3,87.0) ^a^	91.9 (90.1,93.7)	0.233	<0.001	<0.001
	Senior	85.0 (83.2,86.9)	85.5 (83.6,87.6)	77.9 (76.1,79.8) ^b^	90.1 (88.2,91.9)	-	-	-
Starch	Adult	99.6 (99.5,99.7)	99.7 (99.6,99.7)	99.6 (99.6,99.7) ^a^	99.6 (99.5,99.6)	1.000	<0.001	0.001
	Senior	99.6 (99.5,99.7)	99.7 (99.7,99.8)	99.4 (99.3,99.5) ^b^	99.6 (99.5,99.7)	-	-	-
Total dietary fibre	Adult	10.8 (3.69,17.8)	6.64 (−0.65,13.9)	15.9 (8.83,22.9)	16.0 (8.93,32.1)	0.002	0.242	0.138
	Senior	20.6 (13.5,27.6)	26.9 (19.4,34.5)	24.8 (17.7,31.9)	27.7 (20.6,34.8)	-	-	-
Calcium	Adult	0.20 (−7.78,8.18)	−6.69 (−14.9,1.60)	−21.4 (−30.1,−12.8)	−19.3 (−28.4,−10.3)	0.024	0.128	<0.001
	Senior	5.56 (−2.42,13.5)	13.8 (5.13,22.4)	−18.5 (−27.2,−9.89)	−8.34 (−16.3,−0.35)	-	-	-
Phosphorus	Adult	4.87 (−2.09,11.8)	25.9 (20.1,44.1)	21.1 (14.1,28.1)	10.8 (3.83,17.8)	0.314	0.388	<0.001
	Senior	8.18 (1.21,15.1)	36.6 (29.1,44.1)	21.0 (14.1,28.0)	18.9 (11.9,25.9)	-	-	-
Faecal DM%	Adult	36.5 (34.9,38.1)	32.4 (30.8,34.1)	24.1 (22.5,35.7)	37.4 (35.7,39.0) ^a^	0.256	0.008	<0.001
	Senior	34.8 (33.2,36.5)	31.8 (30.1,33.5)	24.0 (22.4,25.6)	33.0 (31.3,34.6) ^b^	-	-	-
Faecal score ^2^	Adult	2.41 (2.13,2.70)	2.55 (2.25,2.84)	2.87 (2.58,3.15)	2.11 (1.82,2.40)	0.995	0.084	<0.001
	Senior	2.67 (2.39,2.96)	2.27 (1.97,2.58)	2.63 (2.34,2.91)	2.00 (1.71,2.28)	-	-	-

LF-LFb, Low-Fat–Low-Fibre; LF-MFb, Low-Fat–Moderate-Fibre; HF-LFb, High-Fat–Low-Fibre; HF-MFb, High-Fat–Moderate-Fibre. ^1^ Different superscripts (^a,b^) denote significant differences (*p* < 0.05) between adult and senior cats within diet. ^2^ Based on a 1–5 scoring system, with increasing values indicating increasing softness in stool quality.

## Data Availability

The datasets analysed during the current study are available from the corresponding author on reasonable request.

## Supplementary Materials

The following are available online at https://www.mdpi.com/article/10.3390/ani11072127/s1, Figure S1: Apparent digestibility (%) of (**a**) crude protein; (**b**) total dietary fibre (TDF); (**c**) calcium; and (**d**) phosphorus in Beagles (*n* = 32) and Brittany (*n*= 5) dogs across four diets with varying levels of crude fibre and total dietary fibre, Figure S2: Apparent digestibility (%) of (**a**) crude protein; (**b**) total dietary fibre (TDF); (**c**) calcium; and (**d**) phosphorus in female (*n* = 27) and male (*n* = 10) dogs across four diets with varying levels of crude fibre and total dietary fibre, and Table S1: Coefficient of variation (CV) in dogs from the same breed, gender, or from the same breed and gender (inter-animal variability) for body weight (BW, kg), daily energy intake (kcal/kgBW^0.75^), crude protein (CP), fat, total dietary fibre (TDF), calcium (Ca), and phosphorus (P) apparent digestibility.
