# Supplementary material for: Healthy Ageing Is Associated with Preserved or Enhanced Nutrient and Mineral Apparent Digestibility in Dogs and Cats Fed Commercially Relevant Extruded Diets
Source: Animals (Basel). 2021 Jul 17;11(7):2127. doi: 10.3390/ani11072127 (PMC8300295; doi:10.3390/ani11072127)
Supplement: Supplementary file 1 [file animals-11-02127-s001.zip › Supplementary Material_Healthy_Ageing_Pets.FigureS2.R1.pdf]

Supplementary Material: Figure S2

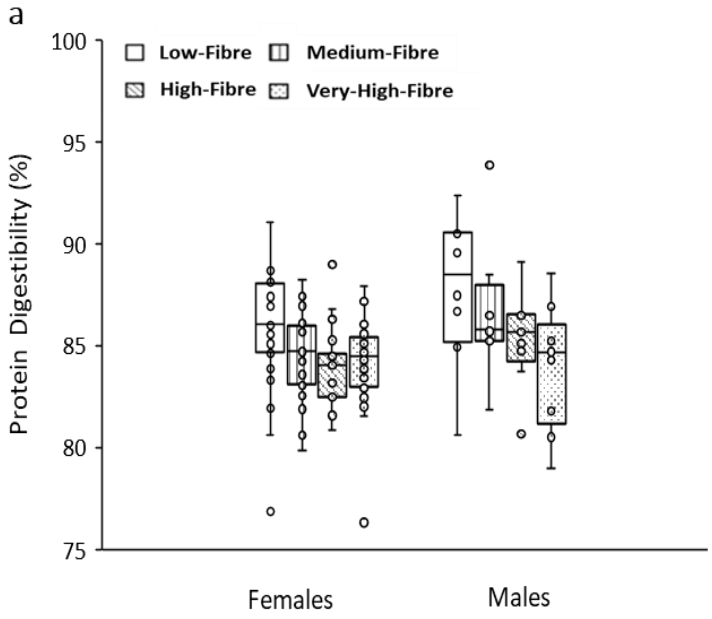

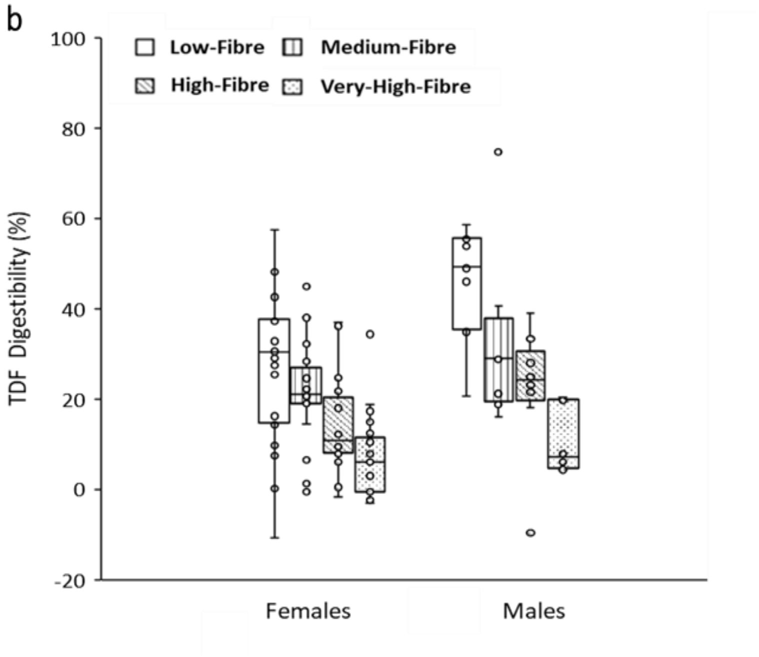

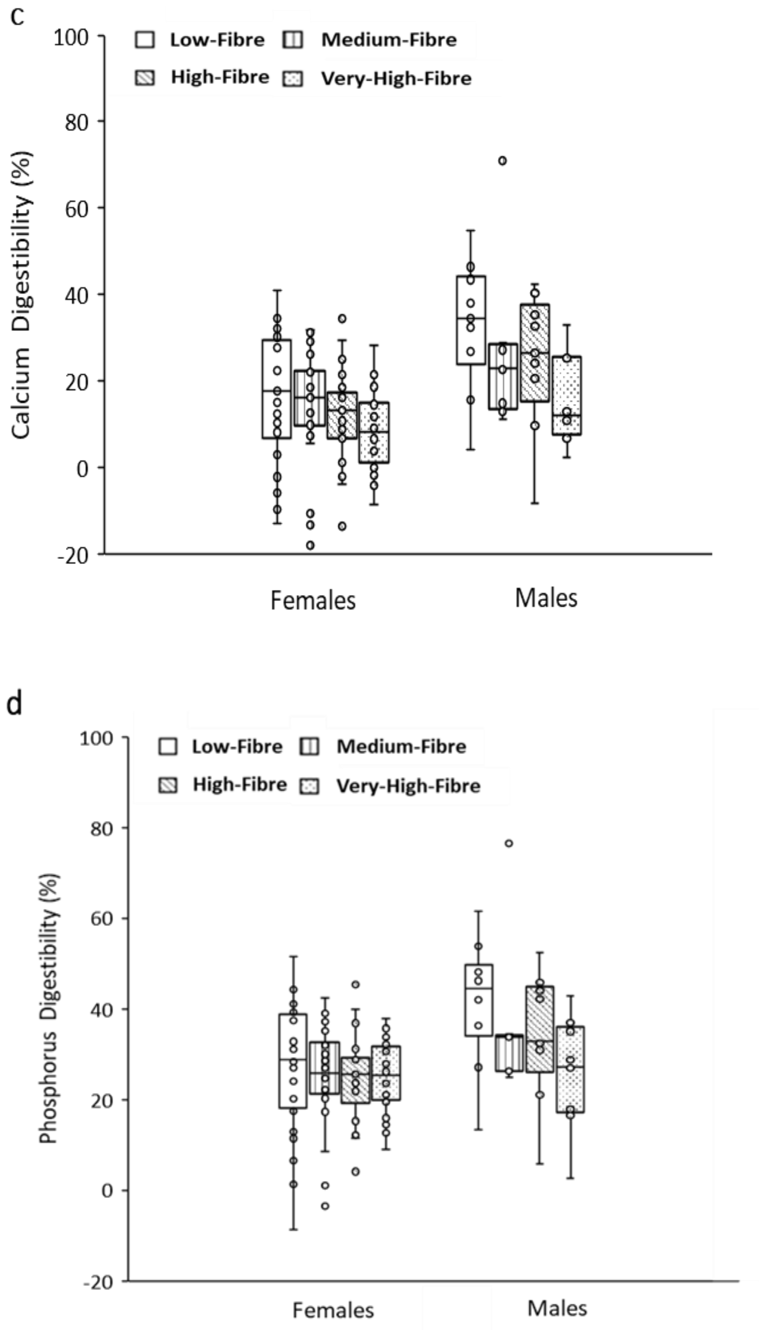

Figure S2. Apparent digestibility (%) of (**a**) crude protein; (**b**) total dietary fibre (TDF); (**c**) calcium; and (**d**) phosphorus in female (n= 27) and male (n= 10) dogs across four diets with varying levels of crude fibre and total dietary fibre
