# Supplementary material for: Healthy Ageing Is Associated with Preserved or Enhanced Nutrient and Mineral Apparent Digestibility in Dogs and Cats Fed Commercially Relevant Extruded Diets
Source: Animals (Basel). 2021 Jul 17;11(7):2127. doi: 10.3390/ani11072127 (PMC8300295; doi:10.3390/ani11072127)
Supplement: Supplementary file 1 [file animals-11-02127-s001.zip › Supplementary Material_Healthy_Ageing_Pets.TableS1.R1.pdf]

Supplementary Material: Table S1

Table S1. Coefficient of variation (CV) in dogs from the same breed, gender, or from the same breed and gender (inter-animal variability) for body weight (BW, kg), daily energy intake (kcal/kgBW$^{0.75}$), crude protein (CP), fat, total dietary fibre (TDF), calcium (Ca), and phosphorus (P) apparent digestibility

			Apparent digestibility (%)				
**CV (%)**	**BW**	**Energy intake**	**CP**	**Fat**	**TDF**	**Ca**	**P**
Beagle (female + male)	17.4	17.4	3.30	2.30	84.7	96.9	46.8
Brittany (female + male)	20.9	15.6	2.45	2.05	59.3	53.8	33.2
**Mean CV (gender differences)**	**19.2**	**16.6**	**2.88**	**2.18**	**72.01**	**75.4**	**40.0**
Females (Beagle + Brittany)	22.0	16.2	2.90	2.17	84.6	97.0	42.4
Males (Beagle + Brittany)	27.6	22.2	3.86	2.56	63.8	62.7	42.6
**Mean CV (breed differences)**	**24.8**	**19.2**	**3.38**	**2.36**	**74.2**	**79.9**	**42.5**
Beagle/Female	14.6	16.1	2.85	2.16	85.7	101	42.8
Beagle/Male	17.4	20.2	4.44	2.81	66.9	64.1	45.1
Brittany/Female	15.1	14.1	3.05	2.40	67.2	50.6	35.2
Brittany/Male	24.3	4.01	2.21	1.94	58.1	57.4	33.8
**Mean CV (inter-animal)**	**17.8**	**13.6**	**3.14**	**2.33**	**69.5**	**68.3**	**39.3**
